# DNA-damage related genes and clinical outcome in hormone receptor positive breast cancer

**DOI:** 10.18632/oncotarget.10886

**Published:** 2016-07-28

**Authors:** Cristina Nieto-Jiménez, Ana Alcaraz-Sanabria, Raquel Páez, Javier Pérez-Peña, Verónica Corrales-Sánchez, Atanasio Pandiella, Alberto Ocaña

**Affiliations:** ^1^ Translational Research Unit, Albacete University Hospital, Albacete, Spain; ^2^ Cancer Research Center, CSIC-University of Salamanca, Salamanca, Spain

**Keywords:** breast cancer outcome, TRIP13, RAD51, GINS1, MCM2

## Abstract

**Background:**

Control of DNA damage is frequently deregulated in solid tumors. Upregulation of genes within this process can be indicative of a more aggressive phenotype and linked with worse outcome. In the present article we identify DNA damage related genes associated with worse outcome in breast cancer.

**Results:**

2286 genes were differentially expressed between normal breast tissue and basal-like tumors, and 62 included in the DNA metabolic process function. Expression of RAD51, GINS1, TRIP13 and MCM2 were associated with detrimental relapse free survival (RFS) and overall survival (OS) in luminal tumors. The combined analyses of TRIP13+RAD51+MCM2 showed the worse association for RFS (HR 2.25 (1.51-3.35) log rank p= 4.1e-05) and TRIP13+RAD51 for OS (HR 5.13 (0.6-44.17) log rank p=0.098) in ER+/HER2- tumors. TRIP13 is amplified in 3.1% of breast cancers.

**Methods:**

Transcriptomic analyses using public datasets evaluating expression values between normal breast tissue and TNBC identified upregulated genes. Genes included in the DNA metabolic process were selected and confirmed using data contained at oncomine (www.oncomine.org). Evaluation of the selected genes with RFS and OS was performed using the KM Plotter Online Tool (http://www.kmplot.com). Evaluation of molecular alterations was performed using cBioportal (www.cbioportal.org).

**Conclusions:**

Expression of DNA metabolic related genes RAD51, GINS1, TRIP13 and MCM2 are associated with poor outcome. Combinations of some of these genes are linked to poor RFS or OS in luminal A, B and ER+HER2- tumors. Evaluation of its predictive capacity in prospective studies is required.

## INTRODUCTION

Breast cancer is an heterogeneous disease with several molecular alterations [1, 2]. This has been characterized with different methods including immunohistochemistry (IHC) or by using transcriptomic analyses [1, 2]. Whole sequencing studies have also shown heterogeneity at a molecular level including mutations, or copy number modifications [3]. In addition, this biological diversity correlates with a different clinical behavior and pattern of relapse, helping to select among different therapeutic strategies [4].

Tumors that express the estrogen receptor and lack HER2 overexpression have been classified by genomic expression as luminal [2], and are treated with anti-hormonal therapy [2, 5]. Among this group those that are enriched with genes associated with proliferation and therefore linked with a slightly worse outcome have been termed luminal B tumors [2]. This subgroup can be identified by the high expression of KI67 measured by IHC [6]. However, although the luminal A group have better outcome, some of these tumors can have different clinical behavior and be associated with a more aggressive phenotype.

Triple negative breast cancer (TNBC) is a molecular subtype defined by the lack of expression of estrogen and HER2 receptors [1]. It accounts for around 15% of tumors and is associated with worse outcome [1, 4]. Among molecular functions that are altered in breast cancer “cell division” and “DNA damage response” are some of the most modified and relevant, particularly in the TNBC subgroup [5]. In this context, genes that participate in the regulation of these two functions can be the target of novel compounds like those directed against PARP such as Olaparib, or novel agents under development against mitotic kinases [7, 8].

In this project we hypothesized that deregulation of genes involved in “DNA damage metabolism” is not restricted to TNBC, and could be present also in other breast cancer subtypes like in estrogen receptor positive tumors. In this context, estrogen receptor positive tumors that overexpress some of these genes could be those that are associated with a more aggressive phenotype.

In the present article by using gene expression analyses we explore genes related to DNA repair mechanism that are deregulated in luminal tumors and that are associated with poor outcome. We identified a set of genes that predict detrimental outcome only in luminal tumors. In addition, the fact that this set is associated with DNA damage suggests that these genes should be evaluated in future studies as potential predictors of efficacy to DNA damaging agents.

## RESULTS

### Identification of metabolic DNA related genes linked to worse outcome

**Figure 1:**
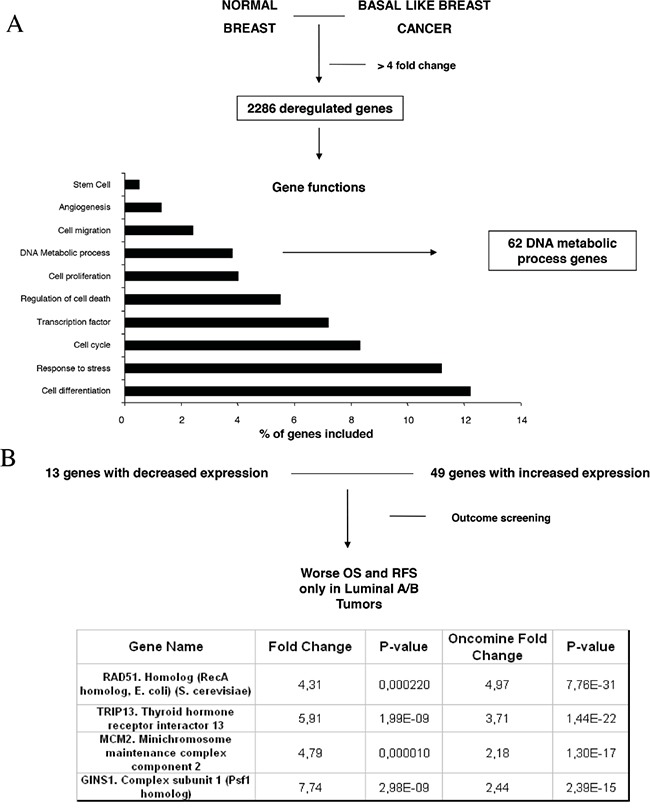
Gene-set enrichment analyses comparing normal epithelial cell with basal like tumors and identification of deregulated genes **A**. Deregulated genes with a minimum of four fold-change difference, and functional analyses of the identified genes, as described in material and methods. **B**. Outcome screening for RFS and OS using KMplotter. Fold changed observed in our dataset and by using data available in Oncomine, as described in material and methods.

To identify functions and relevant genes that could predict worse outcome, we first compared transcriptomic data from normal breast with basal-like tumors using a public dataset (GEO DataSet accession number: GDS2250). Using a cut-off fold change of four or more, we selected 2286 genes differentially expressed between both groups. Functional analyses identified several functions as can be seen in Figure 1A, and we focused on DNA metabolic process where 62 genes were deregulated. The genes included in this function as provided by DAVID bioinformatic resources 6.7 are shown in Table 1. 49 of these genes were upregulated (Figure 1B).

**Table 1:** List of genes deregulated between normal epithelial cells and basal tumors included in the DNA metabolic process as classified by David Bioinformatics Resources 6.7

DAVID Bioinformatics Resources 6.7				
*Term*	*Genes*	*Adjusted p-value (Benjamini)*	*Count/total list*	*Population hits/total*
DNA Metabolic Process (62 different genes)				
DNA Metabolic Process	BRIP1, CDC45L, CHEK1, FANCB, FANCI, GINS1, GINS4, MMS19, RAD51AP1, RAD51, RAD51L1, RAD51L, TRAF3IP2, RECQL4, CIDEA, CDC25A, CDC25C, CDC6, CENPF, CDT1, RMI2, C5orf51, CRY2, CCNE2, CDKN2A, CDKN2C, DSCC1, DTL, ESCO2, ERCC4, EXO1, FOXP1, ELB, HMGB2, HMGA1, IGF1, IGFBP4, KPNA2, LIN9, MND1, MCM10, MCM2, MCM4, MCM6, MSH6, NFIA, NFIB, NCOA6, ORC1L, PTTG1, PTTG3, PCNAPML, RRM2, SMC5, TYMS, TRIP13, TOP2A, USP1, UHRF1, FOS, XPA.	1.0E-01	3.8%	62/2116

As we hypothesized that upregulated genes within the DNA metabolic process could be involved in the oncogenesis of other breast cancer subtypes, we explored next which of the identified genes were associated with worse relapse free survival (RFS) and overall survival (OS) in luminal tumors. We used the KM plotter tool, as described in material and methods, that contains information from datasets grouping more than 3500 patients. As can be seen in Figure 1B, we identified RAD51, GINS1, TRIP13 and MCM2 genes associated with worse outcome in luminal A, and luminal B tumors, for both RFS and OS (Supplementary Table S1, S2). We also confirmed the upregulation of these genes using data available at Oncomine (Figure 1B).

### Gene-set combinations to predict relapse free and overall survival in luminal subtypes

**Figure 2:**
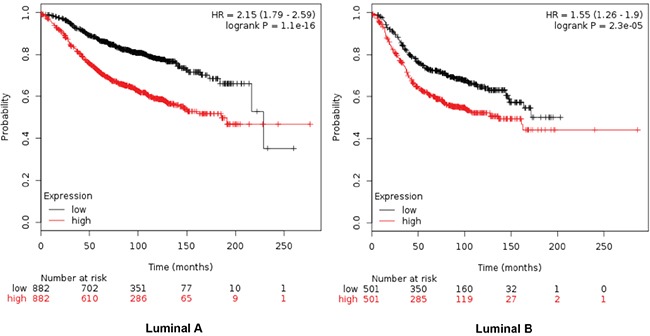
Association of MCM2, TRIP13, GINS1 and RAD51 with RFS in luminal A tumors, and MCM2, TRIP13 and RAD51 for the luminal B subtype

**Figure 3:**
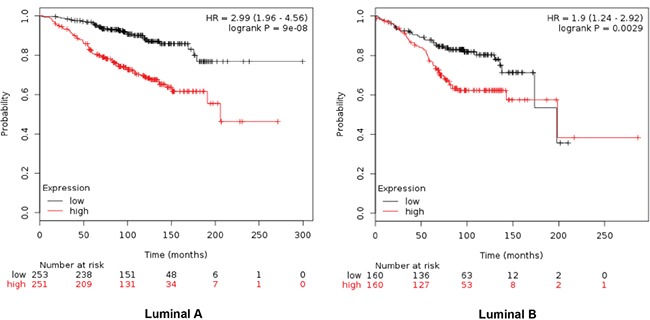
Association of TRIP13 and RAD51 with OS in luminal A tumors, and MCM2, TRIP13 and RAD51 for the luminal B subtype

Combinations of several genes showed stronger prediction for worse outcome that when using genes individually (Table 2). For RFS combination of MCM2, TRIP13, GINS1 and RAD51 showed the worse outcome in luminal A tumors (HR 2.15 (1.79-2.59), log rank p=1.1e-16), meanwhile MCM2, TRIP13 and RAD51 were the best combination to predict outcome in the luminal B subtype (HR 1.55 (1.26-1.9) log rank p= 2.3e-05) (Figure 2). For OS, the best combination was TRIP13 and RAD51 in luminal A tumors (HR 2.99 (1.96-4.56) log rank p=9e-08); and MCM2, TRIP13 and RAD51 in the luminal B subtype (HR 1.9 (1.24-2.92) log rank p=0.0029) (Figure 3).

**Table 2:** List of gene-set combinations in association with RFS and OS in Luminal A and B tumors

Gene Symbols	Overall Survival				Relapse Free Survival			
	Luminal A		Luminal B		Luminal A		Luminal B	
	Hazard Ratio	P-value	Hazard Ratio	P-value	Hazard Ratio	P-value	Hazard Ratio	P-value
**MCM2 + TRIP13**	**1.83 (1.24 - 2.69)**	**2.1E-03**	**1.67 (1.09 - 2.55)**	**0.017**	**1.96 (1.63 - 2.36)**	**2.4E-13**	**1.42 (1.16 - 1.73)**	**7.4E-04**
**MCM2 + GINS1**	**2.06 (1.38 - 3.06)**	**2.8E-04**	**1.45 (0.95 - 2.21)**	**0.08**	**1.98 (1.65 - 2.38)**	**1.1E-13**	**1.28 (1.05 - 1.57)**	**0.016**
**MCM2 + RAD51**	**2.12 (1.43 - 3.14)**	**1.4E-04**	**1.53 (1 - 2.33)**	**0.046**	**1.79 (1.49 - 2.15)**	**1.8E-10**	**1.33 (1.09 - 1.63)**	**5.00E-03**
**TRIP13 + GINS1**	**2.67 (1.76 - 4.06)**	**1.5E-06**	**1.34 (0.88 - 2.04)**	**0.17**	**2.04 (1.7 - 2.46)**	**9.4E-15**	**1.45 (1.18 - 1.77)**	**3.2E-04**
**TRIP13 + RAD51**	**2.99 (1.96 - 4.56)**	**9.00E-08**	**1.76 (1.15 - 2.7)**	**8.2E-03**	**1.93 (1.61 - 2.32)**	**7.8E-13**	**1.52 (1.24 - 1.87)**	**4.4E-05**
**GINS1 + RAD51**	**2.55 (1.69 - 3.86)**	**4.00E-06**	**1.22 (0.81 - 1.85)**	**0.35**	**2.12 (1.76 - 2.55)**	**4.4E-16**	**1.49 (1.22 - 1.83)**	**1.00E-04**
**MCM2 + TRIP13 + GINS1**	**2.18 (1.46 - 3.25)**	**9.4E-05**	**1.59 (1.04 - 2.43)**	**0.03**	**2.13 (1.77 - 2.57)**	**2.2E-16**	**1.45 (1.18 - 1.77)**	**3.3E-04**
**TRIP13 + GINS1 + RAD51**	**2.64 (1.74 - 4.01)**	**2.1E-06**	**1.52 (1 - 2.32)**	**0.05**	**2.1 (1.74 - 2.52)**	**1.00E-15**	**1.32 (1.08 - 1.61)**	**7.2E-03**
**MCM2 + TRIP13 + RAD51**	**2.17 (1.46 - 3.23)**	**9.00E-05**	**1.9 (1.24 - 2.92)**	**2.9E-03**	**2 (1.66 - 2.4)**	**6.4E-14**	**1.55 (1.26 - 1.9)**	**2.3E-05**
**MCM2 + GINS1 + RAD51**	**2.39 (1.6 - 3.59)**	**1.4E-05**	**1.43 (0.94 - 2.18)**	**0.093**	**2.08 (1.73 - 2.5)**	**1.6E-15**	**1.34 (1.09 - 1.64)**	**4.8E-03**
**MCM2 + TRIP13 + GINS1 + RAD51**	**2.34 (1.56 - 3.52)**	**2.4E-05**	**1.57 (1.03 - 2.4)**	**0.035**	**2.15 (1.79 - 2.59)**	**1.1E-16**	**1.46 (1.19 - 1.78)**	**2.6E-04**

### Effect of set combinations on ER+/HER2- patients

**Figure 4:**
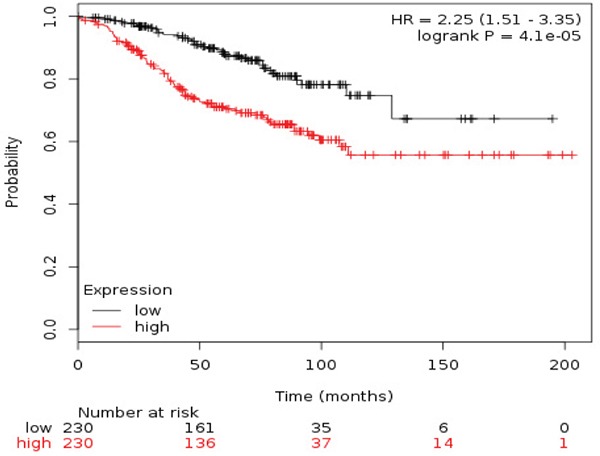
Association of the combined analyses of TRIP13+RAD51+MCM with RFS in ER+/HER2- patients

**Figure 5:**
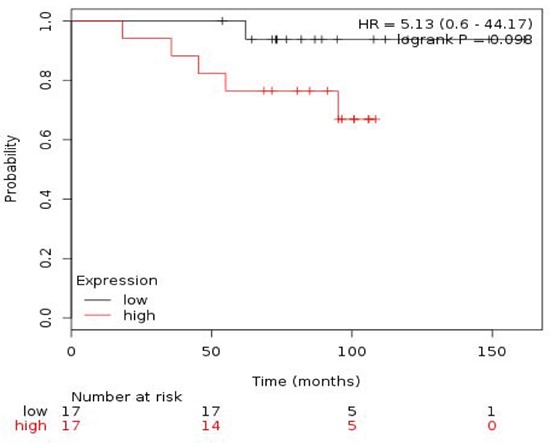
Association of the combined analyses of TRIP13+RAD51 with OS in ER+/HER2- patients

Table 3 described the different combinations in relation to outcome in ER+/HER2- patients. The combined analyses of TRIP13+RAD51+MCM2 showed the poorest association for RFS (HR 2.25 (1.51-3.35) log rank p= 4.1e-05) (Figure 4) and TRIP13+RAD51 for OS (HR 5.13 (0.6-44.17) log rank p=0.098) (Figure 5).

**Table 3:** List of gene-set combinations in association with RFS and OS in ER+/HER2- patients

Overall Survival			Relapse Free Survival		
ER+HER2-			ER+HER2-		
GENE SYMBOLS	Hazard Ratio	P-value	GENE SYMBOLS	Hazard Ratio	P-value
MCM2 + TRIP13	2.14 (0.39 − 11.82)	0.37	MCM2 + TRIP13	2.15 (1.45 − 3.19)	1e−04
MCM2 + GINS1	2.24 (0.41 − 12.29)	0.34	MCM2 + GINS1	2 (1.35 − 2.96)	0.00041
MCM2 + RAD51	0.96 (0.19 − 4.86)	0.96	MCM2 + RAD51	1.92 (1.3 − 2.84)	0.00083
TRIP13 + GINS1	2.24 (0.41 − 12.29)	0.34	TRIP13 + GINS1	2.12 (1.43 − 3.13)	0.00013
TRIP13 + RAD51	5.13 (0.6 − 44.17)	0.098	TRIP13 + RAD51	2.17 (1.47 − 3.21)	7.3e−05
GINS1 + RAD51	2.04 (0.37 − 11.22)	0.4	GINS1 + RAD51	1.81 (1.23 − 2.66)	0.0021
MCM2 + TRIP13 + GINS1	2.14 (0.39 − 11.82)	0.37	MCM2 + TRIP13 + GINS1	2.22 (1.49 − 3.3)	5.6e−05
TRIP13 + GINS1 + RAD51	2.04 (0.37 − 11.22)	0.4	TRIP13 + GINS1 + RAD51	2.21 (1.49 − 3.28)	5.4e−05
MCM2 + TRIP13 + RAD51	1.95 (0.35 − 10.85)	0.44	MCM2 + TRIP13 + RAD51	2.25 (1.51 − 3.35)	4.1e−05
MCM2 + GINS1 + RAD51	2.04 (0.37 − 11.22)	0.4	MCM2 + GINS1 + RAD51	2.06 (1.39 − 3.06)	0.00024
MCM2 + TRIP13 + GINS1 + RAD51	2.04 (0.37 − 11.22)	0.4	MCM2 + TRIP13 + GINS1 + RAD51	2.21 (1.48 − 3.28)	6.3e−05

### Molecular alterations in identified genes and biological functions

Finally we evaluated if the identified genes have any relevant molecular alteration in breast tumors by using information contained at cBioportal [9]. No mutations or deletions were identified in a relevant proportion of patients. Amplification of TRIP13 was observed in 3.1% of the 974 breast invasive carcinoma samples (Table 4). Functions of the genes are described in Supplementary Table S3.

**Table 4:** Copy number alterations (amplifications/deletions) or mutations in the identified genes using data contained in cBioportal as described in material and methods

974 Breast Invasive Carcinoma Samples			
Gene Name	Amplification	Deletion	Mutation
MCM2. Minichromosome maintenance complex component 2	**0.6%**	**0.1%**	**0.3%**
TRIP13. Thyroid hormone receptor interactor 13	**3.1%**	**0.2%**	**0.2%**
GINS1. GINS complex subunit 1 (Psf1 homolog)	**0.7%**	**0.1%**	**0.2%**
RAD51. RAD51 homolog (RecA homolog, E. coli) (S. cerevisiae)	**0.5%**	**0.9%**	**0.2%**

## DISCUSSION

In the present article we describe a set of genes included within the metabolic DNA process that are linked with worse outcome in luminal breast tumors.

Luminal tumors are a breast cancer subtype that expresses hormone receptors and that is usually targeted with hormonal therapy. In addition, it is associated with good clinical outcome. However, not all patients within this group have the same clinical behavior and some have poor outcome. In this context, it is relevant to identify subgroups of patients that express the estrogen receptor and are associated with a detrimental outcome in order to optimize therapeutic strategies. In our article we describe a set of genes that discriminate luminal tumors, identifying a subset linked with poor prognosis.

The DNA repair machinery is usually hyper activated in tumors and in many occasions deficiencies in some of their components have been linked with the genesis and maintenance of some tumors [8]. In TNBC molecular alterations at a genomic level are implicated in the oncogenesis of this tumor and furthermore can help to identify patients that would response to some therapies [5, 8]. In this project we used basal-like tumors as a model to identify genes that were upregulated in this disease. These genes, as demonstrated latter, could also be expressed in other tumor types, and be linked with a more aggressive phenotype.

In our work, we have identified four genes that are associated with poor outcome in luminal tumors. These genes code for different proteins. MCM2 is a DNA replication licensing factor that has been described as associated with worse outcome in ovarian carcinoma [10]. TRIP13 promotes early steps of the DNA double-strand break repair and its presence was associated with progression in prostate cancer [11]; and GINS1 plays a role in the initiation of DNA replication and has been part of a gene-set associated with outcome in early stage non-small cell lung cancer [12]. Finally overexpression of RAD51 has also been linked with poor outcome in colorectal carcinomas [13]. Of note the involvement of these genes in breast cancer has not been fully explored.

Our work has limitations, this is an *in silico* analysis based on functional genomics using data from several sources, so evaluation of these findings in an homogeneous data set is mandatory. In addition, no multivariable analyses is included so it is impossible to identify any potential association with other prognostic factors. Finally, the data should be considered hypotheses generating and be confirmed in independent datasets and in homogenous prospective and retrospective studies.

In conclusion, we describe a gene signature linked with DNA metabolic process that is associated with poor outcome in luminal tumors. Our findings have potential to discriminate patients with higher risk of relapse, therefore helping to customize therapies.

## MATERIALS AND METHODS

### Identification of upregulated genes by transcriptomic analyses

We extracted mRNA level data of normal breast tissue and basal-like cancers from a public dataset (GEO DataSet accession number: GDS2250). Affymetrix CEL files were downloaded and analyzed with Affymetrix Transcriptome Analysis Console 3.0. We selected genes with minimum 4-fold differential expression values between both groups. Gene set enrichment analyses using DAVID Bioinformatics Resources 6.7 was used to analyze the list of genes and to identify functions of these genes (https://david.ncifcrf.gov/), using an adjusted p-value <0.05. Data contained at oncomine (www.oncomine.org) was used to independently confirmed the difference among the selected genes.

### Analyses of outcome for RFS and OS

To evaluate the relationship between the presence of different genes and patient clinical outcome we used the KM Plotter Online Tool (http://www.kmplot.com) in different breast cancer subtypes [14].

This is a public database containing information from 3500 patients that permits to investigate the association of genes with overall survival (OS) and relapse-free survival (RFS).

In this dataset breast cancer subtypes are defined as follow: Triple negative: ER-/HER2-. Luminal A: ESR1+/HER2-/MKI67 low. Luminal B: ESR1+/HER2-/MKI67 high and ESR1+/HER2+ and Basal-like: ESR1-/HER2-.

### Evaluation of molecular alterations

To explore the presence of mutations, deletions or amplifications in the identified genes we used data contained at cBioportal (www.cbioportal.org) [9].

## SUPPLEMENTARY MATERIALS TABLES


