# Supplementary material for: DNA-damage related genes and clinical outcome in hormone receptor positive breast cancer
Source: Oncotarget. 2016 Jul 28;8(38):62834–41. doi: 10.18632/oncotarget.10886 (PMC5609884; doi:10.18632/oncotarget.10886)
Supplement: Supplementary file 1 [file oncotarget-08-62834-s001.pdf]

DNA-damage related genes and clinical outcome in hormone receptor positive breast cancer

SUPPLEMENTARY TABLES

Supplementary Table S1: Association of the identified genes with RFS in the different breast cancer subtypes using KM plotter

See Supplementary File 1

Supplementary Table S2: Association of the identified genes with OS in the different breast cancer subtypes using KM plotter

See Supplementary File 2

Supplementary Table S3: Biological function for the identified genes

See Supplementary File 3
